# Pattern matching for high precision detection of LINE-1s in human genomes

**DOI:** 10.1186/s12859-022-04907-4

**Published:** 2022-09-13

**Authors:** Juan O. Lopez, Jaime Seguel, Andres Chamorro, Kenneth S. Ramos

**Affiliations:** 1 Department of Computer Science and Engineering, University of Puerto Rico, Mayagüez, Puerto Rico; 2 Institute of Biosciences and Technology, Texas A&M Health, Houston, TX USA; 3 Department of Computer Science, University of Puerto Rico, Arecibo, Puerto Rico

**Keywords:** LINE-1, GFF, K-mer, Probe

## Abstract

**Background:**

Long interspersed element 1 (LINE-1 or L1) retrotransposons are mobile elements that constitute 17–20% of the human genome. Strong correlations between abnormal L1 expression and several human diseases have been reported. This has motivated increasing interest in accurate quantification of the number of L1 copies present in any given biologic specimen. A main obstacle toward this aim is that L1s are relatively long DNA segments with regions of high variability, or largely present in the human genome as truncated fragments. These particularities render traditional alignment strategies, such as seed-and-extend inefficient, as the number of segments that are similar to L1s explodes exponentially. This study uses the pattern matching methodology for more accurate identification of L1s. We validate experimentally the superiority of pattern matching for L1 detection over alternative methods and discuss some of its potential applications.

**Results:**

Pattern matching detected full-length L1 copies with high precision, reasonable computational time, and no prior input information. It also detected truncated and significantly altered copies of L1 with relatively high precision. The method was effectively used to annotate L1s in a target genome and to calculate copy number variation with respect to a reference genome. Crucial to the success of implementation was the selection of a small set of k-mer probes from a set of sequences presenting a stable pattern of distribution in the genome. As in seed-and-extend methods, the pattern matching algorithm sowed these k-mer probes, but instead of using heuristic extensions around the seeds, the analysis was based on distribution patterns within the genome. The desired level of precision could be adjusted, with some loss of recall.

**Conclusion:**

Pattern matching is more efficient than seed-and-extend methods for the detection of L1 segments whose characterization depends on a finite set of sequences with common areas of low variability. We propose that pattern matching may help establish correlations between L1 copy number and disease states associated with L1 mobilization and evolution.

## Background

Transposable elements or Transposons, such as the Long Interspersed Element I (L1), Alu and SVA elements, are DNA sequences that move from one location in the genome to another. These elements are important contributors to genome evolution, as well as genetic variation and genomic instability, and are associated with several diseases, including neurofibromatosis, choroideremia, cholinesterase deficiency, Apert syndrome, Dent’s disease, and Walker-Warburg syndrome [1]. L1 is known to be the only active autonomous non-LTR transposon in the human genome. This means that it has the ability to copy and paste itself or other non-autonomous transposons into different genome locations [2, 3], thus boosting its detrimental effects. With more than 500, 000 sequences present, L1s account for 17% of the human genome. L1s consist of two open reading frames, the so-called ORF1 and ORF2, 5’ and 3’ untranslated regions (UTRs), an inter-ORF region, and a poly(A) tail [3]. While most L1s are inactive due to rearrangements, point mutations, and truncation [2, 3], full-length, active L1s can be pathogenic and are the most likely to retrotranspose at significant rates, with at least 124 L1-mediated insertions linked to genetic diseases [3, 4].

Consequently, our method concentrates on full-length L1 sequences. Our main source of data was the L1Base 2 database [5, 6]. Within the Genome Reference Consortium’s human reference genome GRCh38, L1Base 2 reports 146 ORF-intact L1s, 107 ORF2-intact L1s (disrupted ORF1, but intact ORF2), and 13,418 retrotransposition-inactive, full-length non-intact L1s.

### Existing detection tools for the study of mobile elements

We considered some existing tools such as VariationHunter [7], Tea [8], RetroSeq [9], and Tangram [10] for detection of L1s. However, these tools focus on the detection of mobile element insertions, not deletions. This is a considerable limitation since L1 copies may experience loss of segments that range from a few to hundreds of base pairs, and the impact of L1 insertions or deletions on the human genome is not yet known. Hence, these tools were deemed inappropriate for comprehensive L1 detection.

A well-known and commonly used tool is RepeatMasker. Its description says that it is a tool designed to “screen DNA sequences for interspersed repeats and low complexity DNA sequences” [11]. RepeatMasker is thus broader, including different types of retrotransposons, DNA transposons, and other transposable elements. The amplitude in scope comes at the price of loss of accuracy in L1 detection. In a few experiments executed by our team, the reported start positions of the LINE-1s were not correct, and sporadic Alus and Mammalian-wide interspersed repeats (MIRs) were found inside L1s, forcing a post-filtering process to obtain true L1s. Additionally, the last component of a LINE-1 is the poly(A) tail, while RepeatMasker often reports another LINE-1 segment after the poly(A) tail.

### Shortcomings of seed-and-extend

We also examined less specialized tools, like BLAST [12], the classical fast tool for approximate gene alignments. In general terms, we posit that tools based on the seed-and-extend strategy, such as BLAST, are not adequate for precise L1 detection. These tools align short segments of length *k*, known as *k*-mers, with segments within the target sequences. Such alignments are known as seeds, and they are extended heuristically to find a complete similar segment. In the case of L1s, the heuristics of extension produce a large number of false positives. For instance, using BLAST to align an L1 from L1Base2 to genomes in the 1000 Genome Project [13], even with a highly restrictive E-value of $10^{-250}$, returns tens of thousands of results. As such, finding the true L1s requires expensive and time-consuming post-processing of results. This outcome is not surprising as seed-and-extend strategies trade precision for computing time.

### Seed-and-pattern match

In this article we introduce a pattern matching strategy for detecting L1s that achieves adequate precision with reasonable computing time and minimum amount of information provided by the user. Here, pattern matching refers to a well-established computational technique where expressions are tested to determine if they match the constituents of a certain pattern. In contrast to the above-studied seed-and-extend strategies, the strategy being introduced is a seed-and-pattern match strategy that replaces the heuristics of the extend phase of seed-and-extend with pattern matching to diminish the input and post-processing burden while augmenting the precision of detection.

Our algorithm seeds a small fixed set of probes and uses information on the positions of the probes in the query set to decide whether a group of seeds is or is not located within an L1. This avoids the heuristics of extension and its limitations, and provides an efficient way of detecting L1s in the human genome.

## Methods and results

In general terms, the problem being addressed can be stated as follows: Given a query class described by a finite set of sequences that share column ranges with local similarities, called a query set, find all segments in the genome that belong to that class. Transposons and mobile genetic elements, in general, are examples of what we refer to as query sets. Highly conserved segments within these elements, such as genes, are in turn, natural candidates for probes. In our particular case, L1s compose our query class.

As described, the pattern matching strategy uses a small set of *k*-mer segments or probes. The probes are segments with high local similarities to most of the sequences in the query set, as this section will explain the details of how they were generated for our algorithm. The probes are stored in 5’ to 3’ orientation, along with their average offset distance from the beginning of the ORF1. The probes and the information associated with them are calculated only once and then reused in each subsequent application. To classify a segment of the target genome as a member of the query class, the probes are first mapped onto the genome with a fast-mapping algorithm. As in the seed-and-extend methods, we call seed a segment in the target genome that is an approximate match to a probe. As a pattern match, we define a sequence of seeds, among a minimum number of seeds *m*, which coincide with the order and distances of the probes in the query set. The distances between the seeds are measured in base pairs and may vary because of indels or segment losses. To account for these variations, we use an input parameter $t> 0$ that represents a threshold bound. Another input parameter is the minimum number of seeds *m* that are required to conform a pattern match. As with *t*, parameter *m* accounts for segments in the target genome that have lost probes, either partially or completely.

Thus, in symbolic terms, a pattern matching with $m = 3$ is as follows: Let $p_1$, $p_2$ and $p_3$ be probes in the 5’ to 3’ orientation and let $d_1$ be the average distance between $p_1$ and $p_2$ in the sequences of the query set. Similarly, let $d_2$ be the average distance between $p_2$ and $p_3$. Suppose $s_1$, $s_2$ and $s_3$ are the corresponding seeds on the target genome and let $\delta _1$ and $\delta _2$ be the base pair distances between $s_1$ and $s_2$, and $s_2$ and $s_3$, respectively. Then, $s_1$, $s_2$, and $s_3$ are considered a pattern match if and only if $|d_1 - \delta _1| \le t$ and $|d_2 - \delta _2| \le t$.

To use pattern matching for the classification of segments in a genome as members of the L1 class, we took the 146 ORF-intact L1s in L1Base2 as the query set. The probes were extracted by successive refinements, as described below, of the multiple sequence alignment of the sequences in the query set. As multiple sequence alignment problems have no objective mathematical function to optimize, most practitioners modify the computed output manually, or ever perform de novo manual alignments. In our case, the multiple alignment was completed by hand using the bioSyntax [14] highlighting package in the vim editor. We found that although UTRs showed a few similarities within certain ranges, the 5’-UTR segments varied greatly within the first 1000 bases. A similar behavior was observed in the 3’-UTR region. However, the ORF1 and ORF2 regions consistently showed regular behavior. We decided to extract the probes exclusively from the ORFs and we handled ORF1s and ORF2s separately.

The multiple alignment of ORFs showed columns where the highest-occurring nucleotide base was present in at least 95% of the sequences. There were several blocks of 50 columns or more of this kind. Consequently, we took a set of 50-mers from these blocks as pre-candidates for probes. To obtain the actual probes, we filtered this set through a three-step refinement process.

1. To optimize coverage, we kept 50-mers that only mapped to their corresponding ORF within each of the members of the query set, this was done to avoid having probes that mapped to both ORFs.
2. From the set of remaining 50-mers, we selected those that had the smallest number of map hits on the genome, such that we could lower the number of false positives.
3. We selected the subset of all non-overlapping 50-mers from the remaining set.

**Fig. 1:**
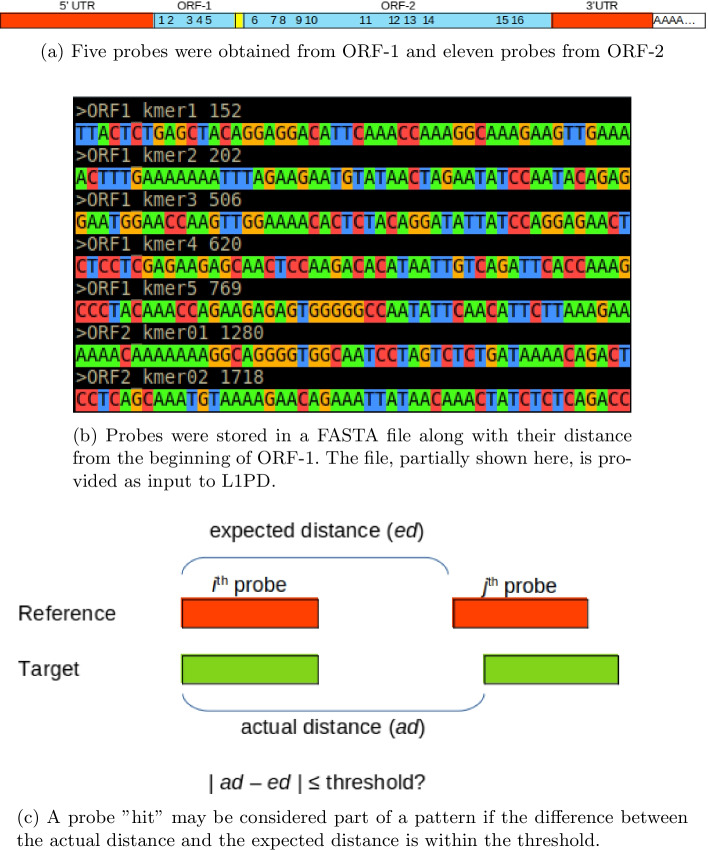
Pattern matching overview

At the end of this filtering process, we were left with five 50-mers from ORF1s and eleven from ORF2s. These sixteen 50-mers were used as our set of probes. Intuitively, this set of probes should provide match patterns strong enough to avoid extensions, but this assumption needed to be verified experimentally. Figure 1 illustrates an overview of the pattern matching strategy.

It should be noted that the values being used (95% similarity, 50 columns, 50-mers) will vary according to the class of sequences that the pattern matching strategy is applied to.

### The LINE-1 pattern detection algorithm

The LINE-1 Pattern Detection (L1PD) algorithm is a computer implementation of the pattern matching strategy for detecting string segments in a given target genome that could be classified as L1s.

The above set of 16 50-mer probes, along with their 5’ to 3’ orientation and average offset distances, were implemented as a lookup table in L1PD. The algorithm maps the probes into the target genome with mrFAST [15, 16], a fast-mapping algorithm that emphasizes the discovery of structural variation and segmental duplications. After seeding the probes, L1PD finds all sequences of *m* or more seeds that conform a pattern match and returns their locations in the target genome.

The input of L1PD were *m*, *t* and $\delta$, as well as the target genome. Its output was the positions within the target genome of all segments classified as L1.

**Fig. 2:**
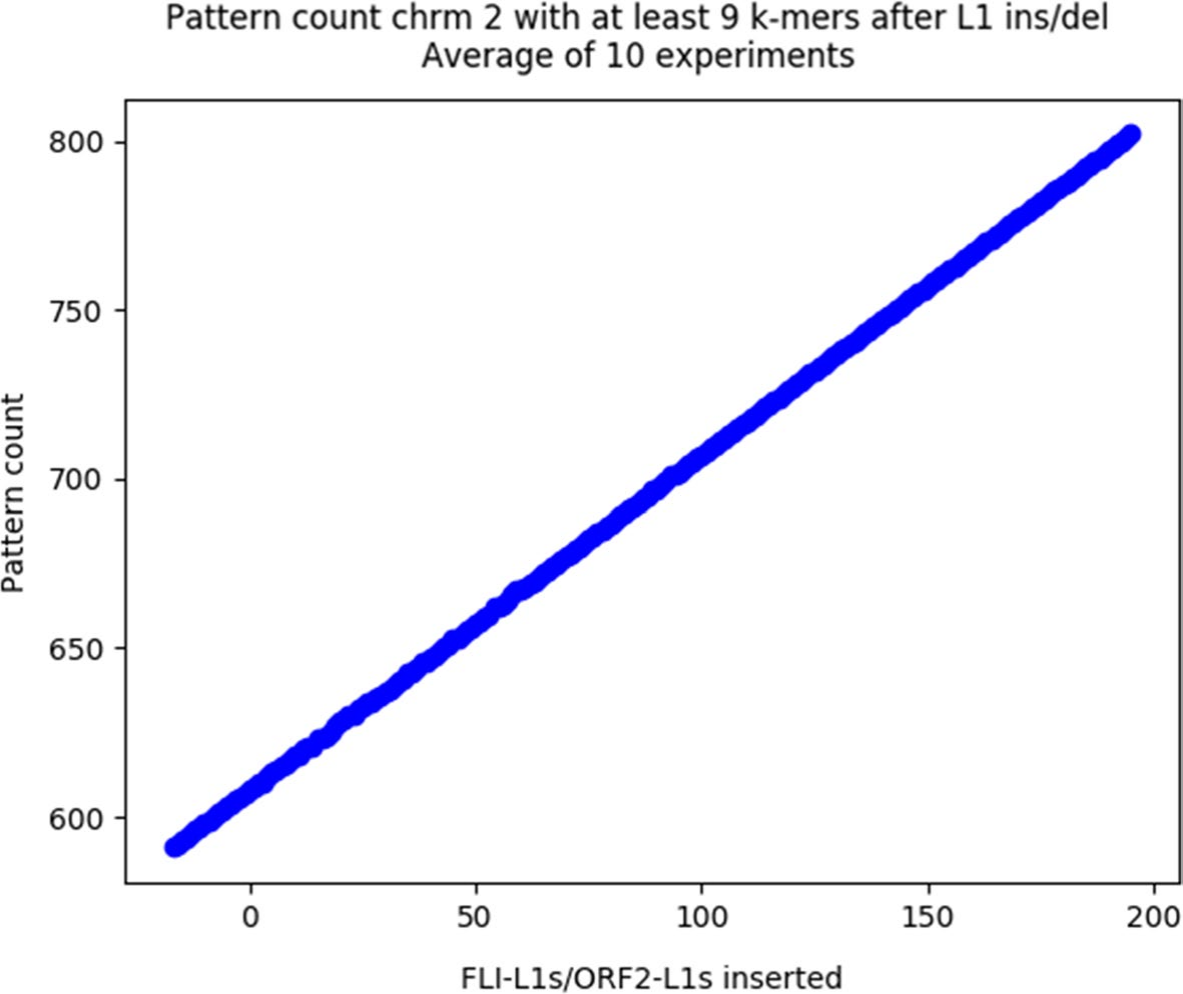
Relationship between random LINE-1 insertion/deletion and pattern count in Chrm 2

**Fig. 3:**
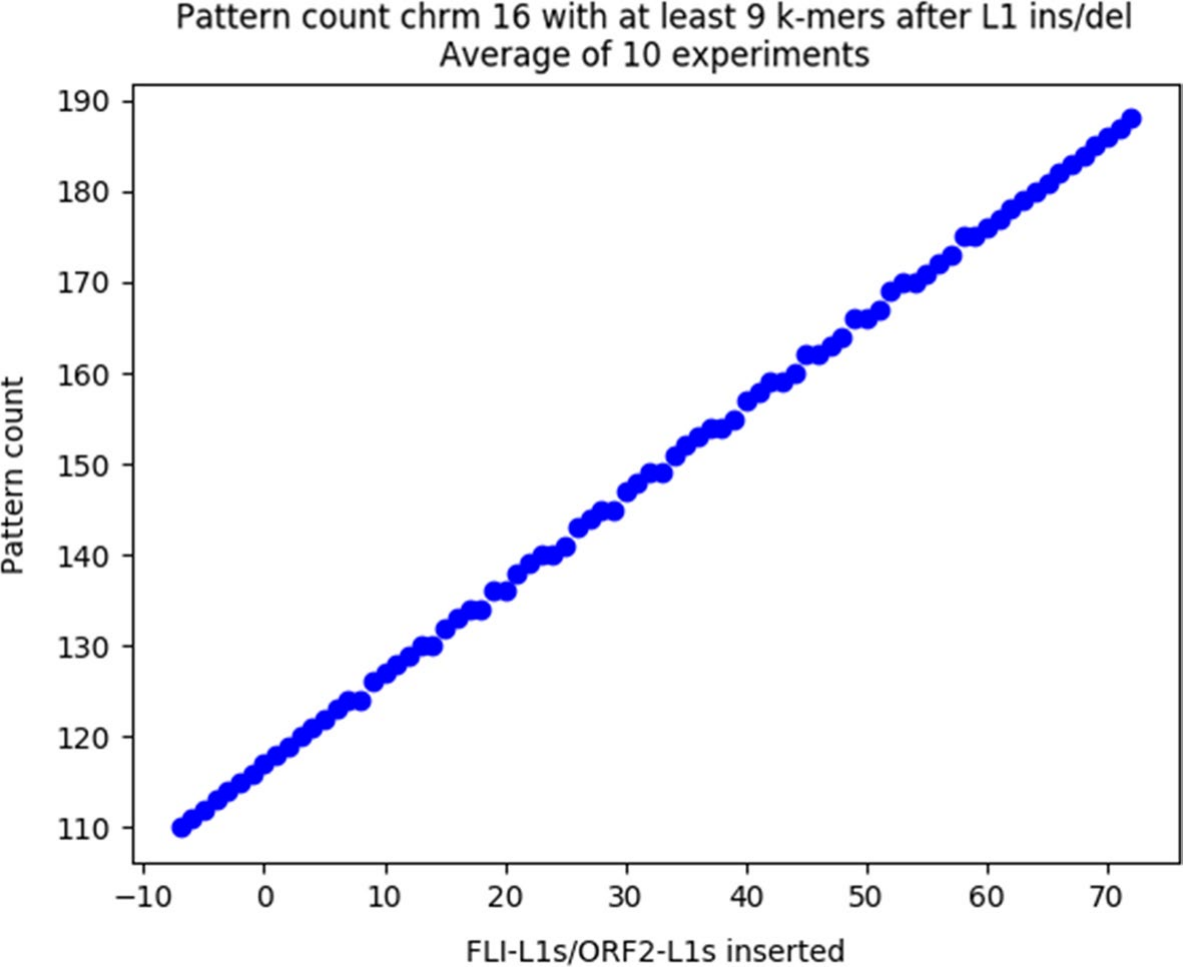
Relationship between random LINE-1 insertion/deletion and pattern count in Chrm 16

We ran a first test with $\delta = 5$, $t = 50$, and *m* ranging from 2 to its maximum of 16. The test aimed to assess the correspondence between pattern matches and L1s achieved with this set of probes. To do this, we randomly added or deleted ORF-intact L1s, and ORF2-intact L1s in different chromosomes of the GRCh38 genome, resulting in L1-altered genomes. We ran L1PD on each of the L1-altered genomes and compared the number of pattern matches and the number of L1s in the modified target. The results are displayed in Figs. 2 and 3. The graphs show a linear correlation between pattern matches and L1 counts, validating the correspondence of pattern matches and L1s.

For $m=2$ to $m=14$, all 146 ORF-intact L1s were accurately detected, but with $m=15$ and $m=16$ one of the ORF-intact L1s was not detected. This ORF-intact L1 had a deletion of about 396 bases in its ORF2. Consequently, the positions to which the probes aligned were shifted by an amount greater than a threshold value of 50 and no pattern match was obtained when $m > 14$.

We further examined the sensitivity of L1PD to its input parameters. We did this in the context of the ability of the method to detect all the L1s in the L1Base2. That is, the 146 ORF-intact, 107 ORF2-intact, and 13,418 non-intact L1s. While it is true that the ORF-intact L1s and ORF2-intact L1s have a major role in the copy and paste activity, a note in the L1Base2 documentation states that non-intact L1s were included because some “may have retained an ability to be expressed and, although at a low frequency, could be retrotransposed by the proteins encoded by retrotransposition-active full length intact L1s” [4]. We assessed this sensitivity in terms of the precision and recall of L1PD outputs.

In information retrieval, the term precision refers to trying to obtain only relevant results (true positives) and minimizing irrelevant results (false positives). On the other hand, the term recall refers to trying to obtain all relevant results (true positives) and not omitting any relevant results (false negatives). The relationship is established by the following equations:

$$\begin{aligned}&\text{ Precision } = \frac{\text{ True } \text{ positives }}{\text{ True } \text{ positives } + \text{ False } \text{ positives }}\\&\text{ Recall } = \frac{\text{ True } \text{ positives }}{\text{ True } \text{ positives } + \text{ False } \text{ negatives }} \end{aligned}$$

In this particular case, precision was calculated as the fraction of true L1s detected by L1PD among all L1s reported by L1PD, using L1Base 2 [5, 6] as reference to determine which were true L1s. Similarly, recall was calculated as the fraction of true L1s detected by L1PD among all L1s present in the genome.

Due to the varying nature of DNA, our algorithm needed to consider possible insertions or deletions, which meant that not all of our probes would be found. Hence, a minimum amount of probe hits in a pattern needed to be established. A higher minimum amount of probes reduced the amount of false positives, as expected, but also reduced the amount of true positives. The net result was that a higher minimum amount of probes resulted in higher precision but lower recall. The F1 Score (also known as the F score or the F measure), based on Van Rijsbergen’s effectiveness measure [17], is the harmonic mean of precision and recall, and is used to establish a balance since it punishes extreme values. The formula is:

$$\begin{aligned} \text{ F1 } \text{ Score } = 2 \times \frac{\text{ Precision } \times \text{ Recall }}{\text{ Precision } + \text{ Recall }}. \end{aligned}$$

The F1 Score is a standard measure that documents classification or query classification performance, and is what we used to determine a set of default values for the input of L1PD.

In mathematical terms, our problem was finding a maximum for the real-valued map

$$\begin{aligned} f(m, t, \delta ) = \mathrm{F1\; Score}, \end{aligned}$$

where $2\le m \le 16$, $25 \le t \le 800$ and $5 \le \delta \le 30$. The boundaries of 800 for *t* and 30 for $\delta$ were defined experimentally. As map *f* takes its values on a finite discrete set, a maximum value can be found by direct computation. As *t* increased within the range of 25 to 700, so did the F1 Score (with one exception where the F1 Score plateaued at $t = 675$, and another where the difference between $t = 675$ and $t = 700$ is negligible). Once *t* went beyond 700, the F1 Score started to decrease (again, with the two aforementioned exceptions). On the other hand, the values of the F1 Score also increased with $\delta$, topping off as $\delta$ neared 50% of the read length. A partial listing of the actual results is shown in Table 1.

Our computations showed that for nearly every value of $\delta$, the maximum F1 Score is obtained when $t = 700$, regardless of the value of *m*. The sole exception was $\delta = 10$, where the maximum F1 Score was achieved at $t = 675$. Hence, we set 700 as the default threshold value. We also found that for almost every value of $\delta$, the highest F1 Score obtained was when $m = 9$. The sole exception was $\delta = 5$, where the highest F1 Score was reached at $m = 7$. Consequently, we set $m=9$ as the default value for L1PD. Finally, after comparing the highest F1 Score obtained for different values of $\delta$, we found that the highest F1 Score overall was obtained when $\delta = 20$. Thus, we set that as the default edit distance for mrFAST in our algorithm.

**Table 1:** F1 Scores of L1PD outputs for different values of parameters *m*, *t*, and $\delta$

$\delta$	*t*	*m*	Precision	Recall	F1 score
5	650	7	0.74503	0.55979	0.63926
	675	7	0.74481	0.56001	0.63932
	700	7	0.74465	0.56016	**0.63935**
	725	7	0.74445	0.56023	0.63932
	750	7	0.74414	0.56038	0.63931
10	625	9	0.79454	0.58554	0.6742
	650	9	0.79409	0.58591	0.67429
	675	9	0.7937	0.5862	**0.67434**
	700	9	0.79321	0.58642	0.67431
	725	9	0.7925	0.58642	0.67405
15	650	9	0.79167	0.58956	0.67582
	675	9	0.7912	0.58986	**0.67585**
	700	9	0.7908	0.59008	**0.67585**
	725	9	0.78995	0.59008	0.67553
	750	9	0.78937	0.59022	0.67541
20	650	9	0.7894	0.59417	0.678
	675	9	0.78893	0.59468	0.67816
	700	9	0.78856	0.59498	**0.67822**
	725	9	0.78774	0.59505	0.67796
	750	9	0.78717	0.5952	0.67785
25	650	9	0.78842	0.59395	0.6775
	675	9	0.78788	0.59447	0.67764
	700	9	0.78745	0.59483	**0.67771**
	725	9	0.78663	0.5949	0.67745
	750	9	0.78606	0.59505	0.67734
30	650	9	0.78819	0.59366	0.67722
	675	9	0.78764	0.59417	0.67735
	700	9	0.78721	0.59454	**0.67743**
	725	9	0.78639	0.59461	0.67718
	750	9	0.78587	0.5949	0.67717

Highest F1 Score for each value of δ is in bold

Table 1 shows some of the values obtained in the search of a maximum F1 Score, where we only included the highest F1 Score for every combination of edit distance and threshold. A more detailed listing may be found in Additional file 1, which may be used to guide users who wish to modify the values of the input parameters either to increase precision or recall. It also establishes the limits of what is achievable by such modifications.

The current version of L1PD takes genomes with their chromosomes assembled as input. Unfortunately, most publicly available genome databases store their genome data in FASTQ files with short-length paired-end reads of a genome. Therefore, for those databases, a time-consuming assembly pipeline was needed to recreate the target genome from the FASTQ files, using GRCh38 as reference. This pipeline includes existing tools such as BWA [18], mrFAST, SAMtools and BCFtools [19], as well as our own scripts. Our scripts were implemented in Python 3 and we used the Biopython [20] module for several intermediate steps during the research, such as generating the k-mer probes.

**Fig. 4:**
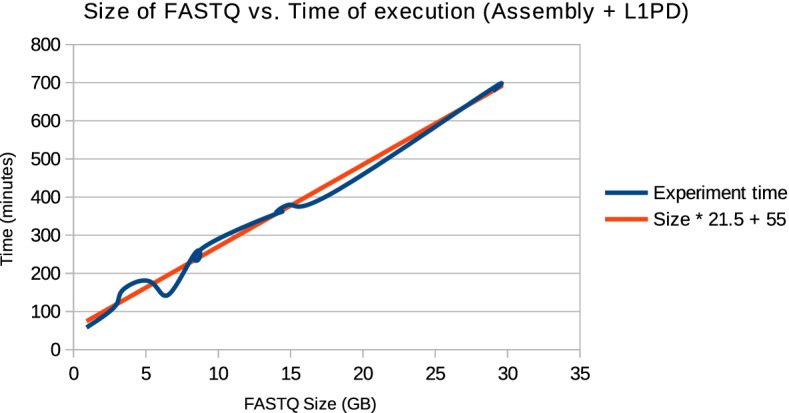
Size vs. Time comparison The time of execution of our pipeline is linearly dependent on the size of the input FASTQ files

The time expenditure required for target genome assembly varies with the size of the FASTQ files containing the reads and can be substantial for large assemblies, as shown in Fig. 4. It is worth noting that once the target genome is assembled the running time of L1PD is constant, as shown in Table 2. Most of the time spent by L1PD is forindexing the target genome and mapping the probes; the time necessary for pattern matching is negligible. Similarly, the memory usage is dictated by the indexing and mapping phases. While using a genome with a size of 3.1GB, indexing used approximately 1.8GB of RAM and mapping used approximately 2GB of RAM.

**Table 2:** Pipeline running time

Sample	FASTQ Size	Pre-processing	L1PD
	(GB)	(min.)	(min.)
HG02153	3.05	93	29
HG00119	3.51	129	28
HG00114	8.03	198	27
HG01383	8.98	235	28
HG00304	9.23	227	28
HG01612	9.70	250	28
HG01883	15.93	354	28
HG01275	19.05	399	28
HG01840	29.33	642	29
HG00551	30.98	686	28

These time and memory measurements, as well as all other runs of L1PD and its applications, were performed on a Dell PowerEdge R740 server, with two Intel^®^ Xeon^®^ Gold 6138 processors. Each processor had 40 cores and ran at 2GHz with a 27.5MB L3 cache. The server had 512GB of DDR4 RAM and 5 SSD SATA Mix hard drives, each with 800GB of storage and speeds of up to 6Gbps. L1PD was coded in Python and the system ran on Ubuntu 18.04.3 LTS.

### Comparisons with BLASR and MUMmer4

Earlier we discussed the shortcomings of the seed-and-extend strategy for the detection of L1s, mentioning BLAST as an example. Two additional examples that use seed-and-extend are BLASR and MUMmer4.

BLASR maps reads that are thousands of bases long, “with divergence between the read and genome dominated by insertion and deletion error” [21]. Since our aim is to detect L1s, in order to provide BLASR with all L1s as the query sequences it was necessary to collect all of the sequences from L1Base2 into a single file. Once this preliminary work was performed, BLASR detected L1s with reasonable accuracy and execution time. Our results were obtained after specifying a minimum percentage of similarity of 100% (using the –minPctSimilarity 100 argument).

MUMmer4 is a genome aligner that was initially designed to align bacterial genomes. This method is capable of handling any genome of biologically realistic length [22]. MUMmer4 consists of two main pipelines, one to align nucleotide sequences (nucmer) and one to align protein sequences (promer). The file with all L1s that was created for use with BLASR was given to nucmer to determine how it would fare finding the L1s. Only 13,671 sequences were being searched for, and although the execution time was reasonable, there were 679,560 results, which is approximately 50 times more than the number of L1s.

L1PD detects L1s with only sixteen 50-mers, which don’t need to be provided as input. We wondered what would be the result of using those same 50-mers as input to BLASR [21] and MUMmer4 [22]. BLASR resulted in 516 hits, which are very few considering there are 16 probes and each should be detected within the 146 ORF-intact L1s ($16 \times 146 = 2,336$). MUMmer4, in turn, returned no results, neither when set to find the Maximal Unique Match (MUM), which represents its default, nor when set to find the Maximal Exact Matches (MEM). Considering that BLASR detected all L1s only when provided with all of the L1 sequences in L1Base2 as input, this experiment clearly shows the high effectiveness of L1PD in detecting L1s with a minimum of information. Table 3 summarizes these results, while Additional file 2 provides more detail.

**Table 3:** L1PD vs. BLASR vs. MUMmer4

	L1PD	BLASR	MUMmer4
Finding L1s in general	Successful with no additional input	Successful with all L1s provided as input	Too many results with all L1s provided as input
Finding L1s with L1PD probes	Successful	Very few results	No results

### L1PD Applications

Next, we present brief descriptions of applications that we implemented on the basis of L1PD.

#### Annotation

L1PD output is generated as a General Feature Format Version 3 (GFF3) file, which is the format used for genome annotations. GFF3 stores genome information features in nine, tab-delimited, text columns. Of the nine columns, L1PD fills the following seven:

- sequence id (chromosome where LINE-1 was found)
- source (“L1PD”)
- type (“mobile_genetic_element”)
- start (start position of the LINE-1)
- end (end position of the LINE-1)
- strand (“+” for forward strand and “-” for reverse strand)
- attributes (“Name=LINE1”)

**Fig. 5:**
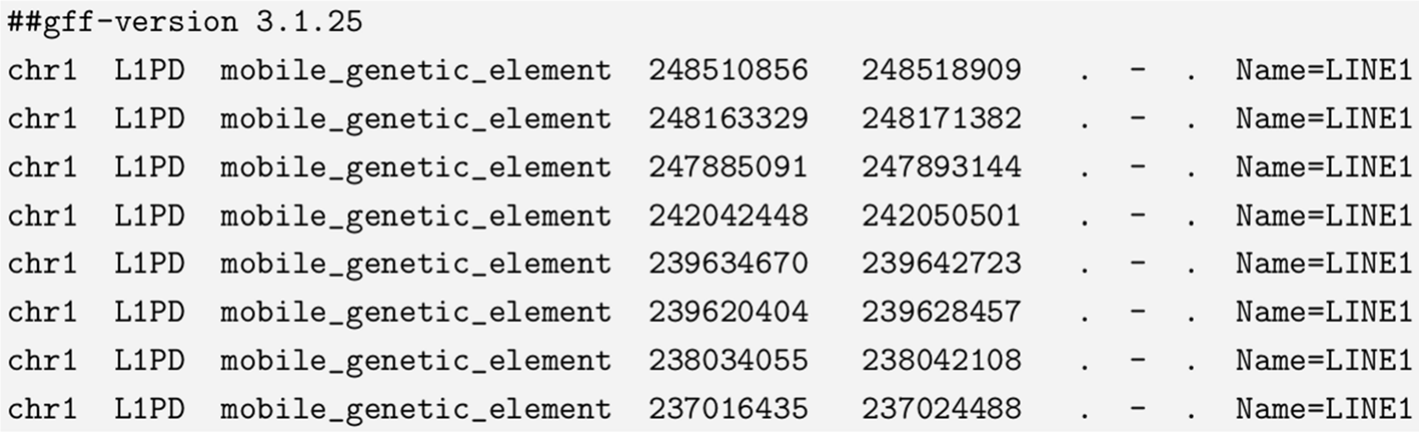
Sample GFF3 output generated by L1PD

In the GFF3 format, the strand field is preceded by the score field followed by the phase field. L1PD does not compute these fields, and therefore, we leave them blank. The GFF3 files generated by L1PD are considered valid by the GFF3 Online Validator [23]. Figure 5 shows a sample of the first lines of an L1PD output file.

#### Copy Number Variations

As discussed earlier, the copy number variation (CNV) of L1s in a target genome may be critical for the diagnosis and prognosis of disease. The reliability of CNV depends on a precise count of L1s, such as the one provided by L1PD. We expressed CNV as a percentage of gain or loss of L1 copies in the GRCh38 genome. We refer to this as CNV percentage of gain (CNVPG), as defined by:

$$\begin{aligned} CNVPG = \frac{SPC - RPC}{RPC} \times 100, \end{aligned}$$

where *SPC* is the subject pattern count and *RPC* is the reference pattern count.

Our application used GCRh38 as reference, but the user can replace this with any genome of interest.

We computed CNVPGs of genomes stored as FASTQ files in the 1000 Genomes Project [13]. Table 4 shows some of the CNVPG values obtained. Positive numbers represent gains, while negative numbers represent losses.

**Table 4:** Sample of CNVPG values

Sample	Pattern count	CNV value
HG02153	8,137	− 0.02457
HG00119	8,139	0
HG00114	8,140	0.01229
HG01383	8,138	− 0.01229
HG00304	8,135	− 0.04915
HG01612	8,128	− 0.13515
HG01883	8,136	− 0.03686
HG01275	8,140	0.01229
HG01840	8,126	− 0.15973
HG00551	8,131	− 0.09829

#### Distribution of LINE-1 insertions

Using the matplotlib Python module we generated a histogram of the L1 counts per chromosome for the target genome and those of GRCh38DH, which is the version of the GRCh38 genome used by the 1000 Genomes Project to account for decoy sequences, alternative haplotypes and Epstein-Barr Virus (EBV).

**Fig. 6:**
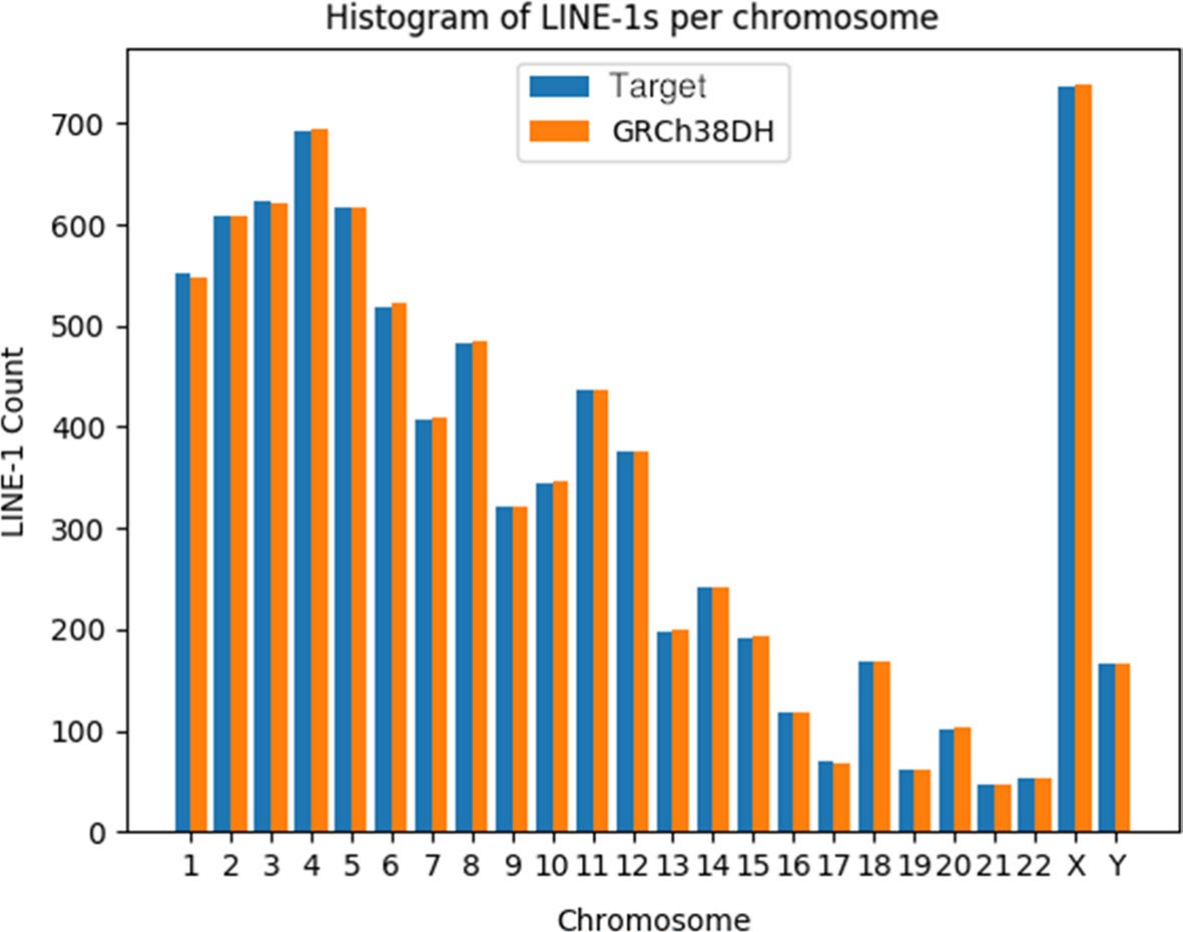
Histogram of LINE-1s per chromosome

Figure 6 shows a histogram in PNG format, generated by L1PD. The *x*-axis in the histogram represents the chromosomes of the reference genome and each bar is the number of L1 copies in that particular chromosome. Besides a visualization of the CNVPG, these histograms serve to also visualize the distribution of L1 insertion sites on the target genome.

**Fig. 7:**
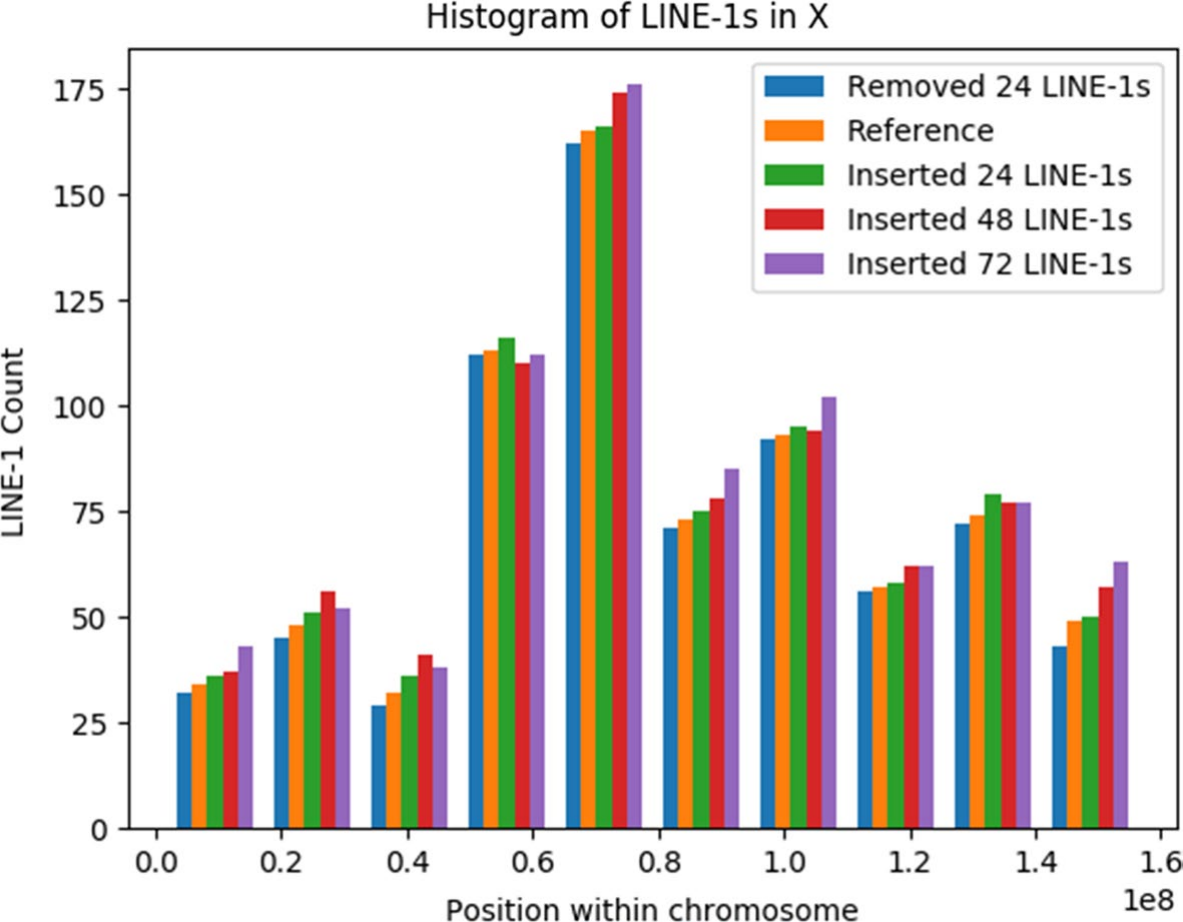
Effect on LINE-1 distribution after random LINE-1 insertion/deletion in Chrm X

Additionally, these histograms are instrumental in the visualization of anomalous L1 counts. Figure 7 compares the L1 counts of a chromosome to which copies of L1s have been randomly removed or added, where the x axis represents the position (offset) within the chromosome where the L1s are detected. This visualization can be particularly useful when searching for correlations between disease phenotype and the number of L1 copies, as would be seen during tumor development and progression.

## Conclusion

An alternative methodology is proposed here to the traditional seed-and-extend strategy to study L1s present in human genomes. We believe that pattern matching is ideally suited for the detection of genome segments sharing common subsequences, but impacted by truncation or gains and losses of segments over the life of the host organism. The proposed strategy sows a set of probe segments onto a target genome and searches for a characteristic pattern in the seeds. In this manner, the heuristics elements associated with seed-and-extension methods can be avoided, with pattern matching taking the place of the extension phase (seed-and-pattern match).

We discussed the results of L1PD, a software implementation of the pattern matching strategy for the identification of L1 segments in target genomes. Our results showed that the selected probes and associated patterns successfully detected all ORF-intact and ORF2-intact L1s. By adjusting the L1PD input parameters of *m*, *t*, and $\delta$, it was possible to detect a larger number of non-intact L1s but this increased the number of false positives (precision vs. recall).

We also examined L1PD as an annotation tool with its GFF3 output, and the use of L1PD in the estimation of L1 copy number variations, as well as the distribution of L1 insertions into a given chromosome.

We also assessed the ability of L1PD to detect changes in the number of L1 copies using synthetic data. Our results validate the use of L1PD as a tool for establishing a reliable correlation between the number of L1 copies and the stages of a L1 evolution, as would be seen during progression of L1-associated diseases.

In summary, the pattern matching strategy can be effectively used for the detection of L1 genome segments. Its implementation in L1PD yielded an economical method in terms of time and computing space. The current running time of the L1PD algorithm is nearly independent of the size of the genome. The performance of the pipeline is, however, affected if it’s necessary to assemble genomes from reads stored as FASTQ files.

The recall of L1PD could be improved by using 75-mer probes instead of 50-mers. Indeed, the use of 75-mers for mrFAST can result in a significant recall improvement, as reported by Phan et al. [24]. Their 50-mer tests gave a recall comparable to the one obtained by L1PD. In our implementation we made the decision to keep 50-mers simply because there are far fewer 75-mers than 50-mers with columns of 95% or more base repeats in the alignment of ORFs in the L1Base2 database. Thus, a change to 75-mers would result in fewer probes, which, in turn, makes it difficult to find patterns that are strong enough to eliminate extensions confidently. It may be possible to work around this problem by relaxing some of the other requirements for the probes. For example, lowering the 95% column-wise similarity to a lower percentage.

mrFAST was used due to its focus on structural variations. However, it is possible to experiment with some of the more recent aligners, such as PuffAligner [25], to see whether improvements can be realized. Additionally, future versions of L1PD may include a VCF/BCF mode so that the user may start off by providing their own .vcf/.bcf files.

### Availability

The source code for L1PD is available under a Creative Commons Attribution-ShareAlike 4.0 license at https://github.com/juan-lopez/L1PD. The code consists of several shell scripts, a Python script, a FASTA file with the probes, as well as sample output files. The shell scripts should run under most Unix-like systems.

L1PD may be executed in one of three modes:

- Genome mode
- BAM/CRAM mode
- FASTQ mode

**Fig. 8:**
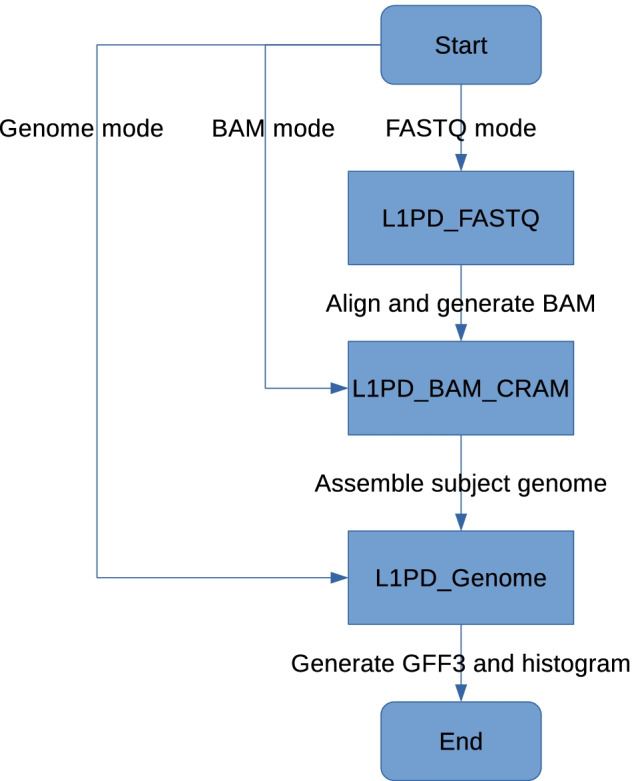
L1PD mode flowchart

BAM/CRAM mode automatically invokes Genome mode, and FASTQ mode automatically invokes BAM/CRAM mode, as shown in Fig. 8.

#### Software requirements and required input

*Genome mode* For Genome mode, which is always executed (directly or indirectly), mrFAST must be installed, as well as Python 3 along with the Matplotlib and Numpy packages. The only required input for L1PD in this mode is the subject genome, although there are other optional inputs available.

*BAM/CRAM mode* BAM/CRAM mode requires BCFtools (version 1.11 or newer) to be installed. The required input for this mode are a BAM/CRAM file and the corresponding reference genome that was used for the alignment, although there are other optional inputs available.

*FASTQ mode* FASTQ mode requires BWA and Samtools (version 1.11 or newer) to be installed. The required input for this mode are paired-end FASTQ files and a reference genome, although there are other optional inputs available.

FASTQ mode is included as an option for users that start out with reads in FASTQ format and who do not have a fully assembled genome. However, the user may opt to use a different reference-free assembly strategy, and then use that assembled dataset in Genome mode.

## Additional file

**Additional file 1.** Determining default values for L1PD.

**Additional file 2.** L1PD vs. BLASR vs. MUMmer4.

## Data Availability

The source code for L1PD is available under a Creative Commons Attribution-ShareAlike 4.0 license at https://github.com/juan-lopez/L1PD.

## Abbreviations

CNV: Copy number variation
CNVPG: Copy number variation percentage of gain
EBV: Epstein-Barr Virus
GFF3: General feature format version 3
L1, LINE-1: Long interspersed element 1
L1PD: LINE-1 pattern detection
MEM: Maximal exact match
MIR: Mammalian-wide interspersed repeat
MUM: Maximal unique match
ORF: Open reading frame
SINE: Short interspersed element
SVA: SINE, VNTR, and Alu
UTR: Untranslated region
VNTR: Variable number tandem repeats
