# Supplementary material for: Pattern matching for high precision detection of LINE-1s in human genomes
Source: BMC Bioinformatics. 2022 Sep 13;23:375. doi: 10.1186/s12859-022-04907-4 (PMC9472350; doi:10.1186/s12859-022-04907-4)
Supplement: Supplementary file 1 — Additional file 1. Determining default values for L1PD. [file 12859_2022_4907_MOESM1_ESM.pdf]

Additional file 1 — Determining default values for L1PD

Some of the key input parameters for L1PD are edit distance, threshold, and minimum amount of probes needed to establish a pattern; these values are denoted by δ, t, and m, respectively. Many different combinations of these values were tested experimentally to try to find the ones that provided the highest F1 Score, and this information was then used to determine the default values for the input parameters. Below are tables that contain more detailed results obtained in the process of determining these default values.

Note that due to the amount of data generated in this process, for each combination of δ and t the only value of m shown is that which resulted in the highest F1 Score. However, in the experiments, m ranged from 2 to 16.

F1 Scores of L1PD outputs for $\delta = 5$ and varying values of m, and t

δ	t	m	Precision	Recall	F1 Score
5	25	7	0.69869	0.48833	0.57486
	50	7	0.71643	0.5132	0.59801
	75	7	0.72286	0.52124	0.6057
	100	7	0.72705	0.52607	0.61043
	125	7	0.72924	0.52936	0.61342
	150	7	0.73234	0.53236	0.61653
	175	7	0.734	0.5347	0.61868
	200	7	0.73543	0.53639	0.62033
	225	7	0.7373	0.53851	0.62241
	250	7	0.73813	0.54019	0.62383
	275	7	0.7393	0.54224	0.62561
	300	7	0.73778	0.54436	0.62647
	325	7	0.73486	0.54597	0.62648
	350	7	0.73527	0.54794	0.62792
	375	7	0.73675	0.54948	0.62947
	400	7	0.73814	0.55094	0.63094
	425	7	0.73866	0.55182	0.63171
	450	7	0.73964	0.55277	0.63269
	475	7	0.74024	0.55365	0.63348
	500	7	0.74083	0.55453	0.63427
	525	7	0.74196	0.55548	0.63531
	550	7	0.74265	0.55643	0.63618
	575	7	0.74429	0.55782	0.63769
	600	7	0.74483	0.55877	0.63851
	625	7	0.74544	0.5595	0.63921
	650	7	0.74503	0.55979	0.63926
	675	7	0.74481	0.56001	0.63932
	700	7	0.74465	0.56016	0.63935
	725	7	0.74445	0.56023	0.63932
	750	7	0.74414	0.56038	0.63931
	775	7	0.7441	0.56045	0.63934
	800	7	0.74383	0.56052	0.63929

F1 Scores of L1PD outputs for $\delta = 10$ and varying values of m, and t

δ	t	m	Precision	Recall	F1 Score
10	25	8	0.71608	0.51934	0.60203
	50	8	0.73209	0.55328	0.63024
	75	9	0.78515	0.53917	0.6393
	100	9	0.78878	0.54553	0.64497
	125	9	0.79088	0.5497	0.64859
	150	9	0.79418	0.55321	0.65214
	175	9	0.79554	0.55614	0.65463
	200	9	0.7968	0.55848	0.65668
	225	9	0.79814	0.56111	0.65895
	250	9	0.79842	0.56294	0.66031
	275	9	0.79942	0.56499	0.66205
	300	9	0.79581	0.56791	0.66281
	325	9	0.78953	0.57047	0.66235
	350	9	0.78836	0.57274	0.66347
	375	9	0.78905	0.57457	0.66493
	400	9	0.78979	0.57603	0.66617
	425	9	0.79058	0.57742	0.66739
	450	9	0.79111	0.57845	0.66826
	475	9	0.79178	0.5794	0.66913
	500	9	0.79231	0.58042	0.67
	525	9	0.79289	0.58137	0.67084
	550	9	0.79328	0.58247	0.67172
	575	9	0.79454	0.58386	0.67309
	600	9	0.79475	0.58488	0.67384
	625	9	0.79454	0.58554	0.6742
	650	9	0.79409	0.58591	0.67429
	675	9	0.7937	0.5862	0.67434
	700	9	0.79321	0.58642	0.67431
	725	9	0.7925	0.58642	0.67405
	750	9	0.79192	0.58657	0.67394
	775	9	0.7917	0.58664	0.67391
	800	9	0.79112	0.58678	0.67379

F1 Scores of L1PD outputs for $\delta = 15$ and varying values of m, and t

δ	t	m	Precision	Recall	F1 Score
15	25	8	0.71312	0.52168	0.60255
	50	9	0.77768	0.53119	0.63122
	75	9	0.78343	0.54246	0.64104
	100	9	0.78718	0.54897	0.64683
	125	9	0.78912	0.55292	0.65023
	150	9	0.79225	0.5565	0.65376
	175	9	0.79362	0.5595	0.6563
	200	9	0.79488	0.56184	0.65834
	225	9	0.79612	0.5644	0.66052
	250	9	0.79639	0.56623	0.66186
	275	9	0.79741	0.56835	0.66366
	300	9	0.79375	0.5712	0.66433
	325	9	0.78745	0.57398	0.66397
	350	9	0.7862	0.57618	0.66499
	375	9	0.78673	0.57801	0.6664
	400	9	0.78755	0.57947	0.66767
	425	9	0.78826	0.58086	0.66884
	450	9	0.78881	0.58196	0.66977
	475	9	0.78942	0.58298	0.67067
	500	9	0.78994	0.584	0.67152
	525	9	0.79052	0.58496	0.67237
	550	9	0.79101	0.58613	0.67332
	575	9	0.79211	0.58752	0.67464
	600	9	0.79239	0.58854	0.67541
	625	9	0.79219	0.5892	0.67577
	650	9	0.79167	0.58956	0.67582
	675	9	0.7912	0.58986	0.67585
	700	9	0.7908	0.59008	0.67585
	725	9	0.78995	0.59008	0.67553
	750	9	0.78937	0.59022	0.67541
	775	9	0.78916	0.5903	0.67539
	800	9	0.78858	0.59044	0.67526

F1 Scores of L1PD outputs for $\delta = 20$ and varying values of m, and t

δ	t	m	Precision	Recall	F1 Score
20	25	8	0.71132	0.52234	0.60235
	50	9	0.77644	0.532	0.63138
	75	9	0.78222	0.54333	0.64124
	100	9	0.7859	0.54992	0.64706
	125	9	0.78792	0.55387	0.65048
	150	9	0.79088	0.55745	0.65395
	175	9	0.79201	0.56045	0.6564
	200	9	0.79311	0.56279	0.65838
	225	9	0.79517	0.56879	0.66318
	250	9	0.79539	0.57069	0.66455
	275	9	0.79631	0.57281	0.66631
	300	9	0.79254	0.57567	0.66691
	325	9	0.78577	0.57845	0.66635
	350	9	0.78417	0.58071	0.66727
	375	9	0.78463	0.58254	0.66864
	400	9	0.78553	0.58408	0.66998
	425	9	0.78614	0.58539	0.67106
	450	9	0.78661	0.58649	0.67196
	475	9	0.78729	0.58752	0.67288
	500	9	0.78784	0.58861	0.6738
	525	9	0.78851	0.58964	0.67472
	550	9	0.78882	0.59073	0.67554
	575	9	0.78991	0.59212	0.67685
	600	9	0.79019	0.59315	0.67763
	625	9	0.78991	0.59381	0.67796
	650	9	0.7894	0.59417	0.678
	675	9	0.78893	0.59468	0.67816
	700	9	0.78856	0.59498	0.67822
	725	9	0.78774	0.59505	0.67796
	750	9	0.78717	0.5952	0.67785
	775	9	0.78688	0.59527	0.67779
	800	9	0.78624	0.59542	0.67764

F1 Scores of L1PD outputs for $\delta = 25$ and varying values of m, and t

δ	t	m	Precision	Recall	F1 Score
25	25	8	0.71092	0.52205	0.60201
	50	9	0.77596	0.53178	0.63107
	75	9	0.78182	0.54312	0.64096
	100	9	0.78551	0.5497	0.64677
	125	9	0.78745	0.55365	0.65016
	150	9	0.79041	0.55723	0.65364
	175	9	0.79154	0.56023	0.65608
	200	9	0.79264	0.56257	0.65807
	225	9	0.7947	0.56857	0.66287
	250	9	0.79492	0.57047	0.66424
	275	9	0.79585	0.57259	0.666
	300	9	0.792	0.57545	0.66657
	325	9	0.785	0.57823	0.66593
	350	9	0.78333	0.58049	0.66682
	375	9	0.78379	0.58232	0.66819
	400	9	0.7847	0.58386	0.66953
	425	9	0.78523	0.58518	0.6706
	450	9	0.7857	0.58627	0.67148
	475	9	0.7863	0.5873	0.67237
	500	9	0.78685	0.58839	0.67329
	525	9	0.7876	0.58942	0.67424
	550	9	0.78791	0.59052	0.67507
	575	9	0.78892	0.5919	0.67635
	600	9	0.78921	0.59293	0.67713
	625	9	0.78893	0.59359	0.67745
	650	9	0.78842	0.59395	0.6775
	675	9	0.78788	0.59447	0.67764
	700	9	0.78745	0.59483	0.67771
	700	9	0.78745	0.59483	0.67771
	725	9	0.78663	0.5949	0.67745
	750	9	0.78606	0.59505	0.67734
	775	9	0.78578	0.59512	0.67728
	800	9	0.78514	0.59527	0.67714

F1 Scores of L1PD outputs for $\delta = 30$ and varying values of m, and t

δ	t	m	Precision	Recall	F1 Score
30	25	8	0.71092	0.52205	0.60201
	50	9	0.77589	0.53156	0.63089
	75	9	0.78173	0.54282	0.64072
	100	9	0.78542	0.54941	0.64654
	125	9	0.78736	0.55336	0.64993
	150	9	0.79032	0.55694	0.65341
	175	9	0.79145	0.55994	0.65585
	200	9	0.79255	0.56228	0.65784
	225	9	0.79462	0.56828	0.66265
	250	9	0.79484	0.57018	0.66401
	275	9	0.79576	0.5723	0.66577
	300	9	0.79192	0.57515	0.66634
	325	9	0.78491	0.57793	0.66569
	350	9	0.78325	0.5802	0.6666
	375	9	0.7837	0.58203	0.66797
	400	9	0.78461	0.58357	0.66931
	425	9	0.78515	0.58488	0.67037
	450	9	0.78562	0.58598	0.67126
	475	9	0.78622	0.587	0.67215
	500	9	0.78676	0.5881	0.67307
	525	9	0.78752	0.58913	0.67402
	550	9	0.78783	0.59022	0.67485
	575	9	0.78876	0.59161	0.6761
	600	9	0.78905	0.59264	0.67688
	625	9	0.7887	0.59329	0.67717
	650	9	0.78819	0.59366	0.67722
	675	9	0.78764	0.59417	0.67735
	700	9	0.78721	0.59454	0.67743
	725	9	0.78639	0.59461	0.67718
	750	9	0.78587	0.5949	0.67717
	775	9	0.78559	0.59498	0.67712
	800	9	0.78494	0.59512	0.67697
