# Supplementary material for: Pattern matching for high precision detection of LINE-1s in human genomes
Source: BMC Bioinformatics. 2022 Sep 13;23:375. doi: 10.1186/s12859-022-04907-4 (PMC9472350; doi:10.1186/s12859-022-04907-4)
Supplement: Supplementary file 2 — Additional file 2. L1PD vs. BLASR vs. MUMmer4. [file 12859_2022_4907_MOESM2_ESM.pdf]

Additional file 2 — L1PD vs. BLASR vs. MUMmer4

L1Base 2 [5, 6] provides three categories of LINE-1s:

1 Full-length intact LINE-1s (FLI-L1s)
2 ORF-2 intact LINE-1s (ORF2-L1s)
3 Full-length non-intact LINE-1s (FLnI-L1s)

There are 146 FLI-L1s, 107 ORF2-L1s, and 13,418 FLnI-L1s. These 13,671 LINE-1s were searched for with L1PD, BLASR, and MUMmer4, in order to be able to compare these strategies. Although L1PD requires no additional input besides the target genome since it is specifically designed for LINE-1s, it was necessary to provide BLASR and MUMmer with input indicating what was being searched for.

Since post-processing on the results for BLASR and MUMmer4 was not performed to filter out false positives, the amount used here for L1PD is also before post-processing, in order to make it a fair comparison.

L1PD vs. BLASR vs. MUMmer4 (without post-processing)

	L1PD	BLASR	MUMmer4
L1s reported using L1Base2 as input	10,315	14,012	679,560

When using only the L1PD probes to search for LINE-1s, the output format of BLASR did not allow for the pattern matching strategy to be directly applied. Hence, only the amount of hits was considered, taking into account that each probe appears once in every FLI-L1.

In this case, BLASR resulted in only 516 hits, but even if only the FLI-L1 are considered, there are 146 FLI-L1 so there should have been approximately 2,336 hits (16 probes $\times$ 146 FLI-L1s). On the other hand, MUMmer4 provided no results.
